# Supplementary material for: Noninvasive urinary protein signatures associated with colorectal cancer diagnosis and metastasis
Source: Nat Commun. 2022 May 19;13:2757. doi: 10.1038/s41467-022-30391-8 (PMC9119985; doi:10.1038/s41467-022-30391-8)
Supplement: Supplementary file 1 — Supplementary Information [file 41467_2022_30391_MOESM1_ESM.pdf]

Supplementary Information

Noninvasive urinary protein signatures associated with colorectal cancer diagnosis and metastasis

Yulin Sun[1], Zhengguang Guo[2], Xiaoyan Liu[2], Lijun Yang[1], Zongpan Jing[1], Meng Cai[1], Zhaoxu Zheng[3], Chen Shao[4], Yefan Zhang[5], Haidan Sun[2], Li Wang[6], Minjie Wang[6], Jun Li[1], Lusong Tian[1], Yue Han[7], Shuangmei Zou[8], Jiajia Gao[1], Yan Zhao[1], Peng Nan[1], Xiufeng Xie[1], Fang Liu[1], Lanping Zhou[1], Wei Sun[2*] & Xiaohang Zhao[1*]

[1]State Key Laboratory of Molecular Oncology, National Cancer Center/National Clinical Research Center for Cancer/Cancer Hospital, Chinese Academy of Medical Sciences and Peking Union Medical College, Beijing, China.

[2]Core Facility of Instruments, Institute of Basic Medical Sciences, Chinese Academy of Medical Sciences and Peking Union Medical College, Beijing, China.

[3]Department of Colorectal Surgery, National Cancer Center/National Clinical Research Center for Cancer/Cancer Hospital, Chinese Academy of Medical Sciences and Peking Union Medical College, Beijing, China.

[4]State Key Laboratory of Proteomics, Beijing Proteome Research Center, National Center for Protein Sciences (Beijing), Beijing Institute of Lifeomics, Beijing, China.

[5]Department of Hepatobiliary Surgery, National Cancer Center/National Clinical Research Center for Cancer/Cancer Hospital, Chinese Academy of Medical Science and Peking Union Medical College, Beijing, China.

[6]Department of Clinical Laboratory, National Cancer Center/National Clinical Research Center for Cancer/Cancer Hospital, Chinese Academy of Medical Sciences and Peking Union Medical College, Beijing, China.

[7]Department of Interventional Therapies, National Cancer Center/Cancer Hospital, Chinese Academy of Medical Sciences and Peking Union Medical College, Beijing, China.

[8]Department of Pathology, National Cancer Center/Cancer Hospital, Chinese Academy of Medical Sciences and Peking Union Medical College, Beijing, China.

[*]Correspondence should be addressed to W.S. (sunwei@ibms.pumc.edu.cn) and X.Z. (zhaoxh@cicams.ac.cn).

Supplementary Figures:

Supplementary Figure 1. Quantitative urinary proteomics analysis in CRC at the discovery stage.

Supplementary Figure 2. The generation of the CRC urinary protein biomarker signature.

Supplementary Figure 3. Independent verification of the urinary protein signature using dot blot analysis for clinical samples.

Supplementary Figure 4. Immunohistochemical analysis of CORO1C, RAD23B and ARPC5 expression in normal and precancerous lesions of CRC.

Supplementary Figure 5. Urinary levels of CORO1C, RAD23B, ARPC5, GSPT2, and NDN in urological tumors.

Supplementary Tables:

Supplementary Table 1. Summary of performance of the biomarkers for CRC diagnosis in PRM verification.

Supplementary Table 2. Summary of performance of the biomarkers for CRC metastasis in PRM validation.

Supplementary Table 3. Summary of performance of the biomarkers for CRC diagnosis in immunoassay verification.

Supplementary Table 4. Summary of performance of the biomarkers for CRC metastasis in immunoassay verification.

Supplementary Table 5. Summary of the biomarkers for CRC diagnosis performance with 95% specificity in immunoassay verification.

Supplementary Table 6. Summary of the biomarkers for CRC metastasis predictive performance with 95% specificity in immunoassay verification.

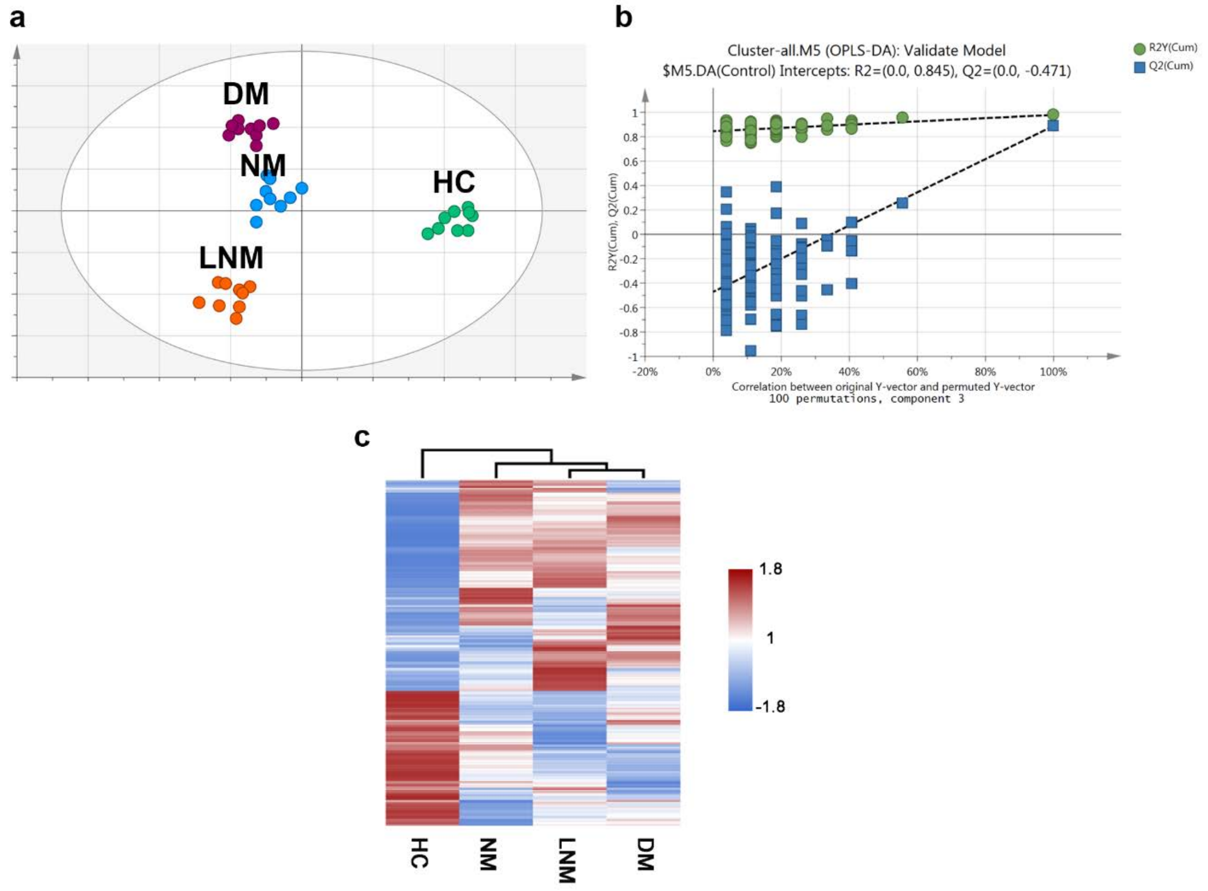

Supplementary Figure 1. Quantitative urinary proteomics analysis in CRC at the discovery stage. (**a**) Score plot of orthogonal partial least squares discriminant analysis (OPLS-DA) model among the four groups. (**b**) One hundred permutation validations of the OPLS-DA model based on the proteome of the four groups. (**c**) Relative abundance of differential proteins among the four groups by unsupervised clustering.

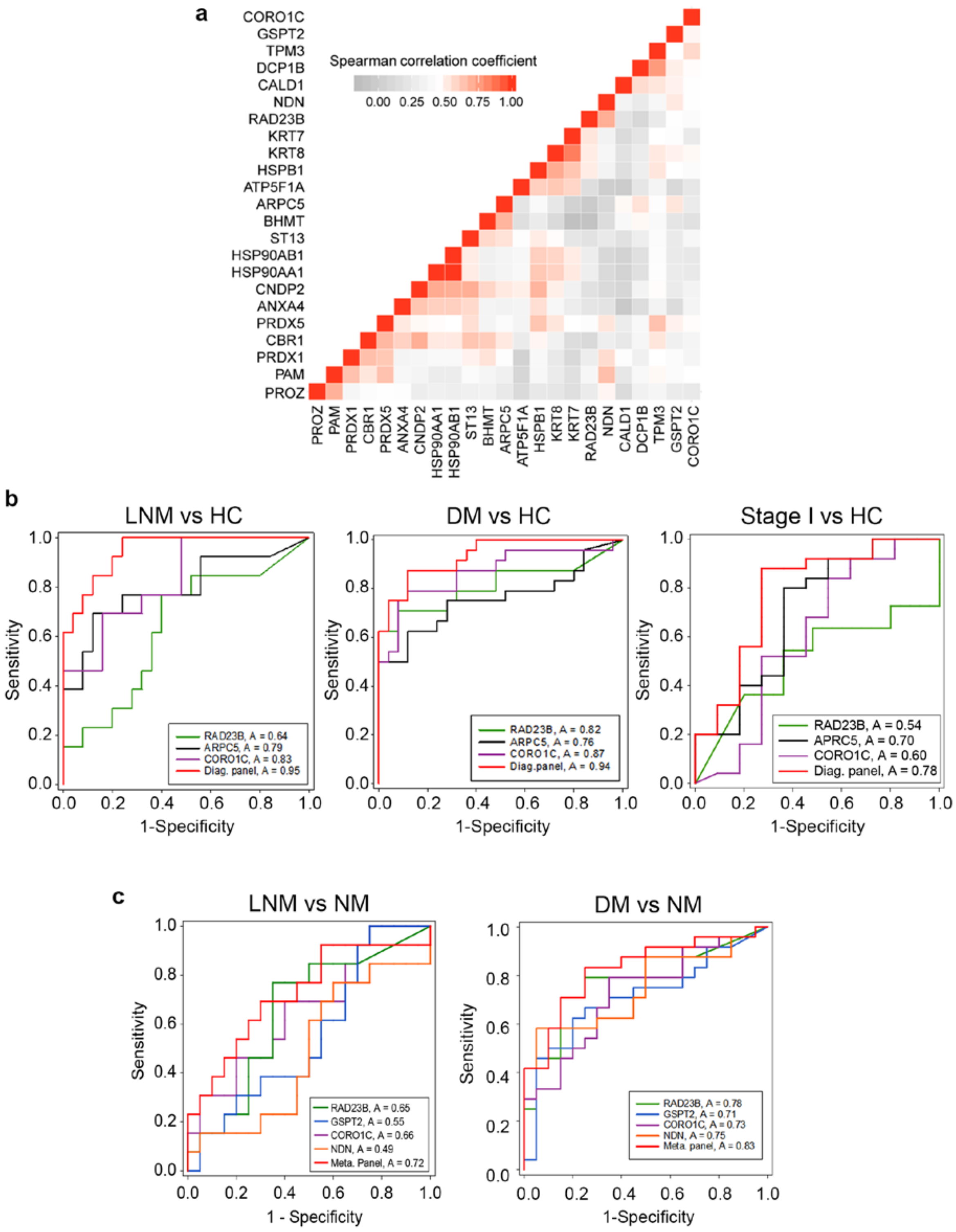

Supplementary Figure 2. The generation of the CRC urinary protein biomarker signature. (**a**) The correlation matrix plot of 23 upregulated proteins in CRC patients based on PRM data. (**b**) ROC curve analysis was conducted by the diagnostic biomarkers to discriminate the HC group from LNM, DM groups and stage I group. (**c**) ROC curve analysis was conducted by the metastatic biomarkers to discriminate the NM group from LNM or DM groups.

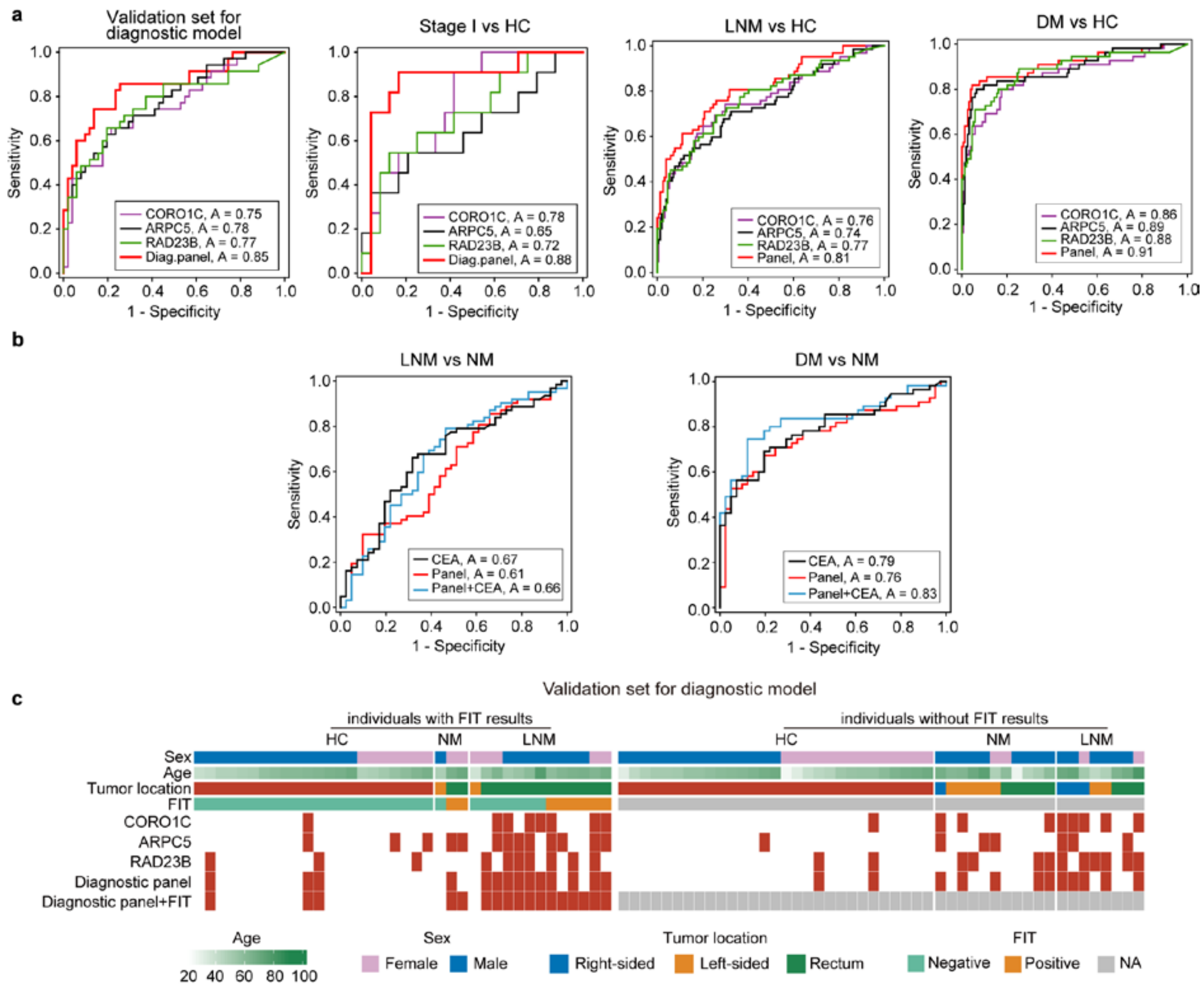

Supplementary Figure 3. Independent verification of the urinary protein signature using dot blot analysis in clinical samples. (**a**) ROC curve of the diagnostic panel and the single markers for the diagnostic model in the validation set, and discrimination between LNM group, DM group or stage I group and HC group. (**b**) ROC curve of serum CEA, the metastatic panel and the combination of the metastatic panel and CEA for the metastatic model showing the discrimination between the LNM group or DM group and NM group. (**c**) Heatmap of the dot plot data for single urinary markers as well as the diagnostic panel, and the combination of FIT test for the diagnostic model in the validation samples with a specificity of 95%. Red: positive using the cutoff value with a specificity of 95%. The FIT test, tumor location, sex and age are indicated by color coding (right side). NA, not available.

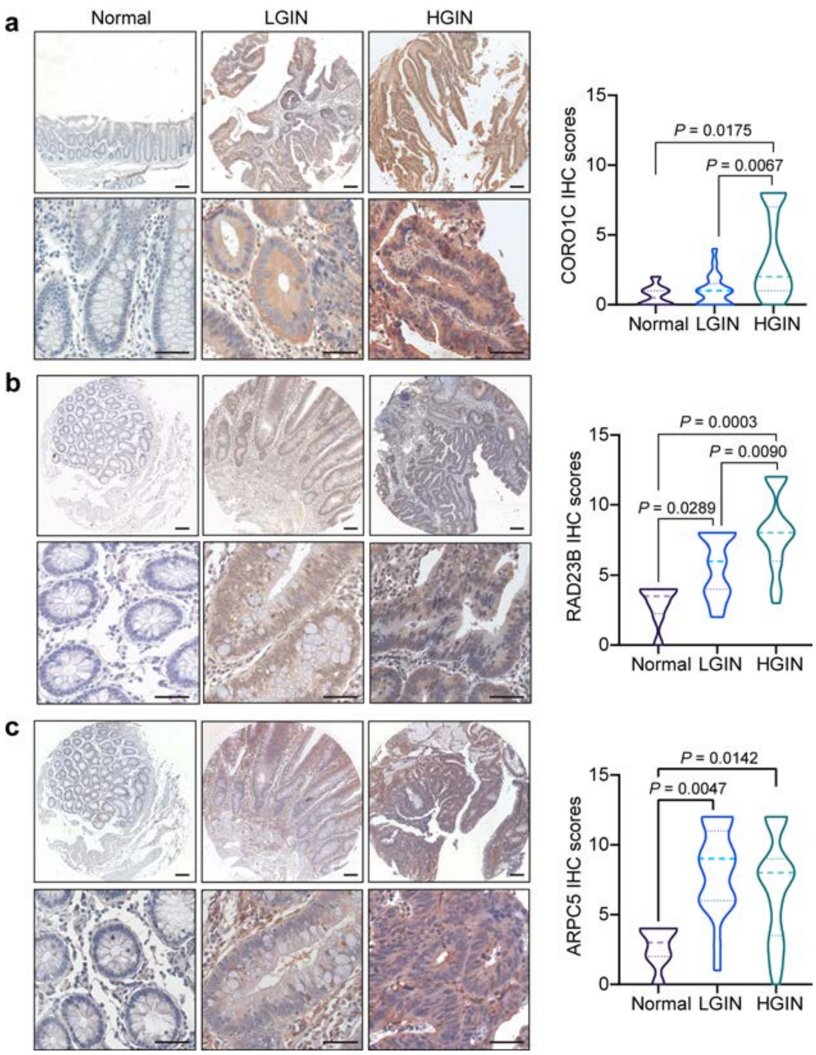

Supplementary Figure 4. Immunohistochemical analysis of CORO1C, RAD23B and ARPC5 expression in normal and precancerous lesions of CRC. Representative immunohistochemistry images and staining scores distribution of CORO1C (**a**), RAD23B (**b**) and ARPC5 (**c**) expression in normal colorectal mucosa, low-grade intraepithelial neoplasia (LGIN) and high-grade intraepithelial neoplasia (HGIN). The differences between groups for each marker were analyzed by Kruskal-Wallis test followed by a Dunn's multiple comparisons test. The median and quartile values in each group of individuals are shown as thick dotted lines and thin dotted lines, respectively. Scale bar: upper panel, 200 μm; bottom panel, 50 μm.

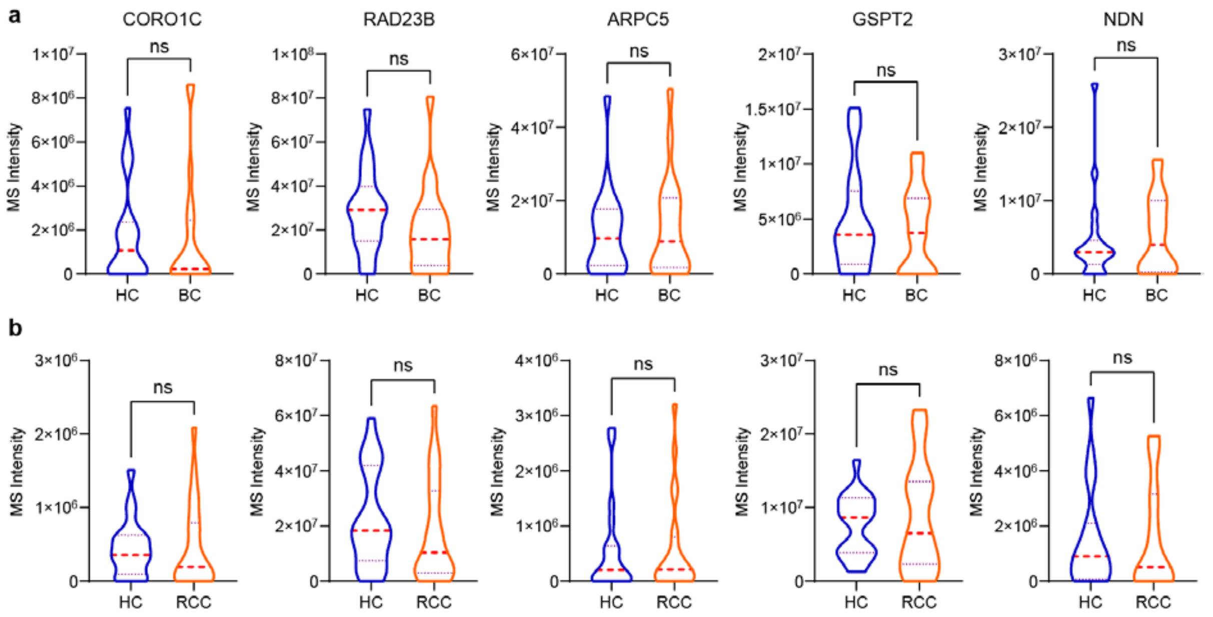

Supplementary Figure 5. Urinary levels of CORO1C, RAD23B, ARPC5, GSPT2, and NDN in urological tumors. The urine samples of patients with bladder cancer (a) and renal cell carcinoma (b) as well as their respective sex- and age-matched healthy controls were measured using a PRM targeted proteomic strategy. The median and quartile values in each group of individuals are shown as thick red dash lines and thin purple dotted lines, respectively. The two-sided Mann-Whitney rank test was used to compare the difference between two groups. HC, healthy controls; BC, bladder cancer; RCC, renal cell carcinoma; ns, not significant.

Supplementary Table 1. Summary of performance of the biomarkers for CRC diagnosis in PRM verification stage.

	CRC-NM vs. HC	CRC-LNM vs. HC	CRC-DM vs. HC	Stage I vs. HC	CRC diagnosis (CRC NM+LNM vs. HC)
CORO1C	0.671	0.828	0.868	0.602	0.733
APRC5	0.724	0.794	0.758	0.702	0.752
RAD23B	0.488	0.637	0.816	0.538	0.547
Diagnostic panel	0.800	0.948	0.935	0.782	0.858

Supplementary Table 2. Summary of performance of the biomarkers for CRC metastasis in PRM validation.

	CRC-LNM vs CRC-NM	CRC-DM vs CRC-NM	CRC metastasis (CRC metastasis vs CRC non-metastasis)
CORO1C	0.662	0.725	0.703
RAD23B	0.650	0.783	0.736
GSPT2	0.554	0.710	0.655
NDN	0.488	0.748	0.657
Metastatic panel	0.723	0.827	0.784

Supplementary Table 3. Summary of performance of the biomarkers for CRC diagnosis in immunoassay verification.

	Training	Validation	NM vs HC	Stage1 vs HC	LNM vs HC	DM vs HC	Training		Validation	
	AUC	AUC	AUC	AUC	AUC	AUC	Sensitivity	Specificity	Sensitivity	Specificity
CORO1C	0.733	0.753	0.713	0.777	0.755	0.857	0.647	0.777	0.657	0.784
ARPC5	0.697	0.776	0.695	0.652	0.741	0.889	0.632	0.718	0.629	0.804
RAD23B	0.728	0.766	0.702	0.723	0.767	0.883	0.676	0.699	0.657	0.804
Diagnostic panel	0.787	0.846	0.796	0.879	0.814	0.913	0.691	0.796	0.743	0.863

Supplementary Table 4. Summary of performance of the biomarkers for CRC metastasis in immunoassay verification.

	LNM+DM vs.NM			LNM vs NM	DM vs NM
	AUC	Sensitivity	Specificity	AUC	AUC
CORO1C	0.635	0.521	0.781	0.566	0.713
RAD23B	0.652	0.769	0.488	0.573	0.742
GSPT2	0.670	0.282	0.976	0.598	0.750
NDN	0.632	0.658	0.610	0.558	0.716
CEA (≥5ng/ml)	NA	0.581	0.780	NA	NA
Metastatic panel	0.699	0.667	0.683	0.610	0.764
Metastatic panel + CEA	0.739	0.709	0.732	0.659	0.831

NA, not available.

Supplementary Table 5. Summary of the biomarkers for CRC diagnosis performance with 95% specificity in immunoassay verification.

	Training		Validation	
	Sensitivity	Specificity	Sensitivity	Specificity
CORO1C	0.182	0.941	0.312	0.954
ARPC5	0.424	0.941	0.625	0.864
RAD23B	0.454	0.941	0.5	0.818
Diagnostic panel	0.515	0.941	0.75	0.818
FIT	0.667	1.000	0.500	1.000
Panel +FIT	0.818	0.941	0.938	0.864

Supplementary Table 6. Summary of the biomarkers for CRC metastasis predictive performance with 95% specificity in immunoassay verification.

	Sensitivity	Specificity
CORO1C	0.137	0.951
RAD23B	0.299	0.951
GSPT2	0.291	0.951
NDN	0.085	0.951
Metastatic panel	0.376	0.951
CEA (≥5ng/ml)	0.581	0.78
Panel + CEA (≥5ng/ml)	0.718	0.756
