# Supplementary material for: Noninvasive urinary protein signatures associated with colorectal cancer diagnosis and metastasis
Source: Nat Commun. 2022 May 19;13:2757. doi: 10.1038/s41467-022-30391-8 (PMC9119985; doi:10.1038/s41467-022-30391-8)
Supplement: Supplementary file 3 — Description of Additional Supplementary Files [file 41467_2022_30391_MOESM3_ESM.pdf]

Description of Additional Supplementary Files

File Name: Supplementary Data 1

Description: The raw TMT quantification data for the four groups (HC group, CRC-NM group; CRC-LNM group; CRC-DM group) with three technical repeats. 9 individual samples were respectively labeled with 126, 127N, 127C, 128N, 128C, 129N, 129C, 130N, and 130C TMT reagents in each group. *: The differential proteins. A criterion of average ratio-fold change greater than 1.5 were used. (.xlxs)

File Name: Supplementary Data 2

Description: IPA Canonical pathway analysis of CRC diagnosis related differential proteins. -Log (*P* value) were shown. The *P* value was calculated using the right-sided Fisher's exact test without adjustments.

File Name: Supplementary Data 3

Description: IPA Disease and Biofunction analysis of CRC diagnosis related differential proteins. -Log (*P* value) were shown. The *P* value was calculated using the right-sided Fisher's exact test without adjustments.

File Name: Supplementary Data 4

Description: IPA Canonical pathway analysis of CRC metastasis related differential proteins. -Log (*P* value) were shown. The *P* value was calculated using the right-sided Fisher's exact test without adjustments.

File Name: Supplementary Data 5

Description: IPA Disease and Biofunction analysis of CRC metastasis related differential proteins. -Log (*P* value) were shown. The *P* value was calculated using the right-sided Fisher's exact test without adjustments.

File Name: Supplementary Data 6

Description: IPA disease and Biofunction analysis of differential proteins in CRC-NM, CRC-LNM, and CRC-DM groups vs. HC group. z-score were shown.

File Name: Supplementary Data 7

Description: IPA Canonical pathway analysis of differential proteins in CRC-NM, CRC-LNM, and CRC-DM groups vs. HC group. -Log (*P* value) were shown. The *P* value was calculated using the right-sided Fisher's exact test without adjustments.

File Name: Supplementary Data 8

Description: PRM validation results of the 41 proteins at the peptide level. (The normalized relative abundance of the proteins in each sample and QC were shown).

File Name: Supplementary Data 9

Description: PRM validation results of the 41 proteins at the protein level. (The normalized relative abundance of the proteins in each sample and QC were shown).

File Name: Supplementary Data 10

Description: Urine creatinine corrected expression intensity of CORO1C, RAD23B, ARPC5, GSPT2 and NDN in 434 cases analyzed by dot blot analysis.

File Name: Supplementary Data 11

Description: Immunohistochemical staining scores of CORO1C in TMAs of CRC.

File Name: Supplementary Data 12

Description: Immunohistochemical staining scores of RAD23B in TMAs of CRC.

File Name: Supplementary Data 13

Description: Immunohistochemical staining scores of ARPC5 in TMAs of CRC.

File Name: Supplementary Data 14

Description: Diagnostic and metastatic biomarkers in previous studies. Biomarkers identified in urine, serum/plasma, or tumor tissue were annotated. *, Diagnostic/ metastatic biomarkers.

File Name: Supplementary Data 15

Description: PRM validation results of the CORO1C, RAD23B, ARPC5, GSPT2, and NDN in bladder cancer patients and their age/sex matched healthy controls (HC).

File Name: Supplementary Data 16

Description: PRM validation results of the CORO1C, RAD23B, ARPC5, GSPT2, and NDN in renal cell carcinoma patients and their age/sex matched healthy controls (HC).
