# Supplementary material for: Noninvasive urinary protein signatures associated with colorectal cancer diagnosis and metastasis
Source: Nat Commun. 2022 May 19;13:2757. doi: 10.1038/s41467-022-30391-8 (PMC9119985; doi:10.1038/s41467-022-30391-8)
Supplement: Supplementary file 20 — Reporting Summary [file 41467_2022_30391_MOESM20_ESM.pdf]

nature research

Corresponding author(s): Xiaohang Zhao and Wei Sun

Last updated by author(s): Mar 15, 2022

Reporting Summary

Nature Research wishes to improve the reproducibility of the work that we publish. This form provides structure for consistency and transparency in reporting. For further information on Nature Research policies, see our Editorial Policies and the Editorial Policy Checklist.

Statistics

For all statistical analyses, confirm that the following items are present in the figure legend, table legend, main text, or Methods section.

n/a	Confirmed	
☐	☒	The exact sample size (n) for each experimental group/condition, given as a discrete number and unit of measurement
☐	☒	A statement on whether measurements were taken from distinct samples or whether the same sample was measured repeatedly
☐	☒	The statistical test(s) used AND whether they are one- or two-sided<br>*Only common tests should be described solely by name; describe more complex techniques in the Methods section.*
☒	☐	A description of all covariates tested
☐	☒	A description of any assumptions or corrections, such as tests of normality and adjustment for multiple comparisons
☐	☒	A full description of the statistical parameters including central tendency (e.g. means) or other basic estimates (e.g. regression coefficient) AND variation (e.g. standard deviation) or associated estimates of uncertainty (e.g. confidence intervals)
☐	☒	For null hypothesis testing, the test statistic (e.g. F, t, r) with confidence intervals, effect sizes, degrees of freedom and P value noted<br>*Give P values as exact values whenever suitable.*
☒	☐	For Bayesian analysis, information on the choice of priors and Markov chain Monte Carlo settings
☐	☒	For hierarchical and complex designs, identification of the appropriate level for tests and full reporting of outcomes
☒	☐	Estimates of effect sizes (e.g. Cohen's d, Pearson's r), indicating how they were calculated

Our web collection on statistics for biologists contains articles on many of the points above.

Software and code

Policy information about availability of computer code

Data collection	The DDA mass data was acquired using xCalibur3.1 from thermofisher scientific. The PRM data was acquired using Analyst2.0 from AB Sciex.
Data analysis	Proteome Discoverer software suite 2.1; Skyline 20.1; SIMCA 14.0; MetaboAnalyst web tool; GraphPad Prism 7; SigmaPlot 14.0; MedCalc 15; IBM SPSS software v18; The source code of caret (6.0-90) and randomForest (4.6-14) packages of R program (4.0.3 and 4.1.2) as well as readme files were deposited to the GitHub website (https://github.com/scshaochen/PRMColonCancer)

For manuscripts utilizing custom algorithms or software that are central to the research but not yet described in published literature, software must be made available to editors and reviewers. We strongly encourage code deposition in a community repository (e.g. GitHub). See the Nature Research guidelines for submitting code & software for further information.

Data

Policy information about availability of data

All manuscripts must include a data availability statement. This statement should provide the following information, where applicable:

- Accession codes, unique identifiers, or web links for publicly available datasets
- A list of figures that have associated raw data
- A description of any restrictions on data availability

The authors declare that the data supporting the findings of this study are available within the Supplementary Information and Supplementary Data files. And Source Data are also provided with this paper. The data of proteomics have been deposited to the Integrated Proteome Resources (iProX, http://iprox.cn) with the dataset identifier IPX0002679000.

Field-specific reporting

Please select the one below that is the best fit for your research. If you are not sure, read the appropriate sections before making your selection.

- [x] Life sciences
- [ ] Behavioural & social sciences
- [ ] Ecological, evolutionary & environmental sciences

For a reference copy of the document with all sections, see nature.com/documents/nr-reporting-summary-flat.pdf

Life sciences study design

All studies must disclose on these points even when the disclosure is negative.

Sample size

No sample-size calculation was performed in this study. We used all of available samples that we collected. Because the sample size is enough large, they are sufficient.

Data exclusions

For urine samples from colorectal cancer patients, we excluded 26.7% of patients with post-operation; a pathological diagnosis with nontubular adenocarcinoma (mucinous adenocarcinoma, melanoma, signet ring carcinoma, neuroendocrine carcinoma); accompanied with other benign or malignant tumors; abnormal renal functions; receiving chemoradiotherapy; a failure of quality control of PRM (without signals in more than 40% of peptides) or dot blot analysis (CV>20%). The enrollment criteria for healthy control subjects were as follows: (1) the absence of benign or malignant tumors; (2) a qualified physical examination finding no dysfunction of vital organs and (3) normal renal function and without albuminuria; (4) a failure of quality control of PRM (without signals in more than 40% of peptides) or dot blot analysis (CV>20%).

The modeling analysis of dot blot data excluded 101 HCs and 21 CRC patients because their serum CEA measurements were not available.

Replication

The DIA analysis (2D-LC-MS/MS) were triple replicated consecutively within one day. 96% of all the proteins performed technical CV are less than 30%.

In PRM analysis, each samples were run twice consecutively within one day. 97% of all the peptides performed technical CV are less than 30%.

In addition, we performed DIA analysis on urine sample and further validated using PRM. These results were reproducible.

Randomization

Block randomization method was used to spilt samples into training and validation sets.

Blinding

The investigators were grouped according to clinical diagnosis: CRC-NM, CRC-LNM, CRC-LM and healthy controls, therefore no blinded analysis was performed.

Reporting for specific materials, systems and methods

We require information from authors about some types of materials, experimental systems and methods used in many studies. Here, indicate whether each material, system or method listed is relevant to your study. If you are not sure if a list item applies to your research, read the appropriate section before selecting a response.

Materials & experimental systems

n/a	Involved in the study	
☐	☒	Antibodies
☒	☐	Eukaryotic cell lines
☒	☐	Palaeontology and archaeology
☒	☐	Animals and other organisms
☐	☒	Human research participants
☒	☐	Clinical data
☒	☐	Dual use research of concern

Methods

n/a	Involved in the study	
☒	☐	ChIP-seq
☒	☐	Flow cytometry
☒	☐	MRI-based neuroimaging

Antibodies

Antibodies used

anti-CORO1C (Cat. No. H00023603-M02, Abnova; 1:1000 dilution; mAb, clone 1F7), anti-ARPC5 (Cat. No. sc-166760, Santa Cruz Biotech., Dallas, TX, USA; 1:1000 dilution, mAb, clone C-3), anti-RAD23B (Cat. No. A1034, ABclonal Technology, Woburn, MA, USA; 1:1000 dilution; rabbit pAb), anti-GSPT2 (Cat. No. 12989-1-AP, Proteintech Group Inc., Rosemont, IL, USA; 1:1000 dilution; rabbit pAb) and anti-NDN (Cat. No. sc-101224, Santa Cruz Biotech; 1:1000 dilution; mAb, clone 36-V) for immunoassay (dot blot analysis).

anti-CORO1C (Cat No. TA349821; OriGene Technologies, Inc, Rockville, MD, USA; 1:50 dilution; rabbit pAb), anti-RAD23B (Cat No. A1034; 1:20 dilution) or anti-ARPC5 (Cat. No. sc-166760; 1:80 dilution) antibodies were used for immunohistochemical staining (IHC).

Validation

All primary antibodies were used according to the manufacturer's instructions for Western blotting (including dot blotting) and IHC.

Validation statements on the manuacturer's website:

anti-CORO1C (H00023603-M02) http://www.abnova.com/products/products_detail.asp?catalog_id=H00023603-M02

anti-CORO1C (TA349821) https://www.origene.com/catalog/antibodies/primary-antibodies/ta349821/coronin-3-coro1c-rabbit-polyclonal-antibody

anti-ARPC5 (sc-166760) https://www.scbt.com/p/p16-arc-antibody-c-3
anti-RAD23B (A1034) https://abclonal.com.cn/catalog/A1034
anti-GSPT2 (12989-1-AP) https://www.ptglab.com/products/GSPT2-Antibody-12989-1-AP.htm
anti-NDN (sc-101224) https://www.scbt.com/zh/p/ndn-antibody-36-v?requestFrom=search
In addition, we had validated all of five antibodies used for dot blot analysis by Western blot in the cell lysates of colorectal cancer. All of bands are all single and specific.

Human research participants

Policy information about studies involving human research participants

Population characteristics

The age, sex, number and detailed clinical information of urine samples in each experiment is shown in Table 1. The age and sex distributions were basically balanced among the HCs and three groups of CRC patients, except that the samples of HC group in the dot blot analysis were much younger. In addition, except for TNM staging, there were basically no statistically significant differences in the clinical parameters of histological differentiation grade, CA19-9 level and tumor location among the three CRC groups (NM, LMN and DM) in the TMT and PRM analysis; however, CEA level, CA19-9 level and tumor location showed significant difference among the three CRC groups in the dot blot analysis.
In addition, the age, sex and detailed clientele-pathological information of immunohistochemistry staining assay are provided in the Supplementary Data 11-13 and Fig. 5b.

Recruitment

For urine samples, A total of 359 CRC patients (242 male and 117 female; median age 59 years, min-max: 26-87 years) were recruited from the Cancer Hospital, Chinese Academy of Medical Sciences, from January 2015 to October 2018. All patients were pathologically diagnosed by two senior pathologists, and random morning midstream urine samples were collected prior to surgical operations or chemotherapy/radiotherapy. We excluded 26.7% of patients with postoperative disease; a pathological diagnosis of nontubular adenocarcinoma (mucinous adenocarcinoma, melanoma, signet ring carcinoma, neuroendocrine carcinoma); other benign or malignant tumors; abnormal renal functions; receiving chemoradiotherapy; and a failure of quality control of PRM (without signals in more than 40% of peptides) or dot blot analysis (CV>20%). The 263 qualified patients were divided into CRC patients without metastases (NM, n = 76), with lymph node metastasis (LNM, n = 97) and with distant metastasis (DM, n = 90) according to the pathology report.
And 298 urine samples from healthy controls (173 male and 125 female; median age 55 years, min-max: 23-78 years) were obtained from the Health Medical Center of the Cancer Hospital and PLA General Hospital from August 2014 to October 2018. The enrollment criteria for healthy control subjects were as follows: (1) the absence of benign or malignant tumors; (2) a qualified physical examination finding no dysfunction of vital organs and (3) normal renal function and without albuminuria. Nine HCs were excluded for quality control of PRM (without signals in more than 40% of peptides) or dot blot analysis (CV>20%).
In the 434 urine samples detected by immunoassay, serum CEA measurements were available in 312 samples, including samples from 154 HCs and 158 CRC patients. To facilitate the comparison of CEA results, these 122 samples (101 HCs and 21 CRC patients) were excluded from the further model analyze.

Ethics oversight

This study was approved by the Ethics Committee of Institute of Basic Medical Sciences and Cancer Hospital, Chinese Academy of Medical Sciences (No. 047-2019, Beijing, China). Because our study was a retrospective non-interventional study, the ethics committee granted an informed consent waiver.

Note that full information on the approval of the study protocol must also be provided in the manuscript.
